# Video Ocular Counter-Roll (vOCR): Otolith-Ocular Function and Compensatory Effect of the Neck Following Vestibular Loss

**DOI:** 10.1002/ohn.304

**Published:** 2023-03-02

**Authors:** Yuchen Yang, Jing Tian, Jorge Otero-Millan, Michael C. Schubert, Amir Kheradmand

**Affiliations:** 1 Department of Neurology, The Johns Hopkins University School of Medicine, Baltimore, Maryland, USA; 2 Department of Otolaryngology–Head and Neck Surgery, The Johns Hopkins University School of Medicine, Baltimore, Maryland, USA; 3 Herbert Wertheim School of Optometry and Vision Science, University of California, Berkeley, California, USA; 4 Department of Physical Medicine and Rehabilitation, The Johns Hopkins University School of Medicine, Baltimore, Maryland, USA

**Keywords:** neck proprioception, ocular counter-roll, otolith, vestibular recovery, video-oculography, vOCR

## Abstract

**Objective.:**

Assessment of recovery following vestibular loss has been limited by the lack of bedside measures in clinical settings. Here, we used the video ocular counter-roll (vOCR) test to study otolith-ocular function and compensatory effect of neck proprioception in patients at different stages of vestibular loss.

**Study Design.:**

Case-control study.

**Setting.:**

Tertiary care center.

**Methods.:**

Fifty-six subjects were recruited including patients with acute (9 ± 2 days [mean ± standard error of mean]), subacute (61 ± 11 days), and chronic (1009 ± 266 days) unilateral loss of vestibular function, as well as a group of healthy controls. We used a video-oculography method based on tracking the iris for vOCR measurement. To examine the effect of neck inputs, vOCR was recorded during two simple tilt maneuvers in all subjects while seated: 30° head-on-body tilt and 30° head-and-body tilt.

**Results.:**

The vOCR responses evolved at different stages following vestibular loss with improvement of the gains in the chronic stage. The deficit was more pronounced when the whole body was tilted (acute: 0.08 ± 0.01, subacute: 0.11 ± 0.01, chronic: 0.13 ± 0.02, healthy control: 0.18 ± 0.01), and the gain of vOCR improved when the head was tilted on the body (acute: 0.11 ± 0.01, subacute: 0.14 ± 0.01, chronic: 0.13 ± 0.02, healthy control: 0.17 ± 0.01). The time course of vOCR response was affected as well with reduced amplitude and slower response in the acute stage of vestibular loss.

**Conclusion.:**

The vOCR test can be valuable as a clinical marker to measure vestibular recovery and compensatory effect of neck proprioception in patients at different stages following loss of vestibular function.

Vestibular inputs converge broadly with other sensory information across various brain functions. These multimodal contributions range from control of gaze and posture to higher-level mechanisms that mediate spatial orientation and motion perception. At the level of vestibulo-ocular reflex (VOR), there is a close interaction between the vestibular signals and the proprioceptive inputs that encode the position of the neck.^1,2^ The neural pathways that mediate these interactions are not well understood, but physiological observations suggest that the brain can adapt to perturbations in vestibular function by using inputs from the neck. These inputs can substitute vestibular signals to improve gaze stabilization and postural control following vestibular loss, highlighting the value of neck inputs as a potential clinical marker to measure and track recovery.^2–7^

To evaluate the effect of neck proprioception following vestibular loss, previous studies have primarily used angular rotations of the head and trunk or applied vibration on the neck muscles.^7–12^ Only a few have directly examined the contribution of neck inputs to otolith-ocular function or corresponding vestibular recovery related to VOR in the roll plane.^13,14^ In particular, such focused assessments of vestibular function could not be done in clinical settings, as the bedside measures of vestibular functions were lacking previously. In recent years, however, portable video-oculography (VOG) has made it possible to quantify VOR examination at the bedside.^15,16^ Video head impulse testing (vHIT) is now widely adopted to measure semicircular canal function and the video ocular counter-roll (vOCR) has been recently introduced as a clinical test of otolith-ocular function.^17–19^

The vOCR test is performed by simply tilting the head in the roll plane. When the head is tilted laterally to the shoulder, the resulting VOR is a torsional rotation of the eyes in the opposite direction of the head tilt (ie, the ocular counter-roll or OCR).^20^ During the movement of the head, the dynamic component of vOCR response is driven by the activity from both the otoliths and semicircular canals, and it consists of torsional nystagmus with a slow phase in the opposite direction of the head tilt.^21,22^ During a sustained head tilt, however, the static component of vOCR that maintains ocular torsion is generated by the otolith inputs and primarily the utricles.^20,23,24^ The gain of static vOCR response is normally about 0.2, but it is significantly reduced in patients with loss of vestibular function.^19,21,25^ The vOCR deficit is more severe in acute patients compared to those with chronic vestibular loss,^18^ suggesting that the vOCR test can be valuable as a clinical marker of recovery in patients with loss of vestibular function.

In the current study, we examined the effect of neck inputs on vOCR responses and asked whether it can be used clinically to assess recovery in patients with vestibular loss. To evaluate the effect of neck inputs, we used a maneuver with the head tilted on the body to measure vOCR and compared the results with the whole-body tilt. Both the dynamic and static components of vOCR response were examined in different stages following vestibular loss. We also compared vOCR results to vHIT as a measure of semicircular canal function in the same groups of patients.

## Methods

Prospectively, we measured vestibular function using vOCR and vHIT in patients with unilateral vestibular loss and healthy controls. Forty-one patients with unilateral loss of vestibular function from resection of vestibular schwannoma (Table 1) were recruited in our study. Based on the previously published data on vOCR measurements,^18,19^ the sample size required for the expected effect size was less than 20 subjects (*α* = .05 and 1 −*β* = 0.8). A healthy control group was also recruited (n = 15; 7 females). The control group had no known neurological or vestibular disorders. Based on the time from surgery, patients were divided into 3 subgroups: acute (within 4 weeks after surgery, n = 13; 6 females), subacute (between 4 weeks and 6 months after surgery, n = 13; 7 females), and chronic (more than 6 months after surgery, n = 15; 8 females) (Table 1). Patients were enrolled consecutively from the Johns Hopkins outpatient clinic or during their hospital stay. The patient recruitment was only based on unilateral loss of vestibular function regardless of whether or not they had any vestibular symptom or sign of vestibular recovery. Those with any other neurological abnormality (eg, ischemic lesions on the brain magnetic resonance imaging) or history of vestibular or hearing loss on the nonlesion side were not included. Four patients were excluded due to difficulty completing the maneuvers after the surgery. All subjects gave written informed consent, and the study was approved by the institutional review board at Johns Hopkins.

### Experimental Procedures

#### vOCR Test

**Figure 1.**
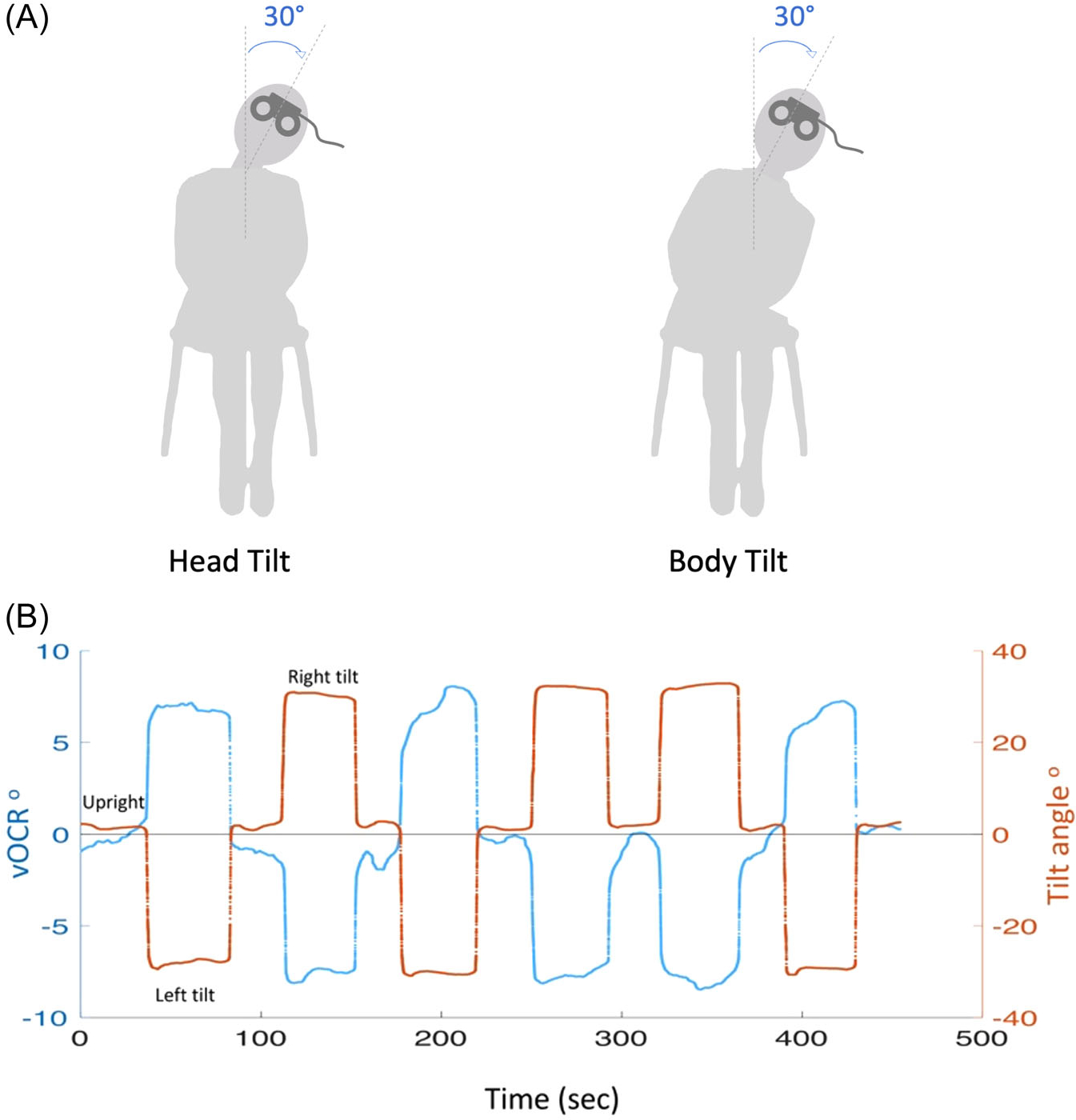
(A) For vOCR recording, the head tilt maneuver was a passive 30° lateral tilt of the head on the trunk and the body tilt maneuver was a passive 30° lateral tilt of the head and trunk en bloc. In each maneuver, the recording was started in the upright position followed by 3 right and 3 left tilts in random orders separated by a return to the upright position, during which vOCR and tilt angle were measured simultaneously. (B) An example of vOCR recording (blue trace/left axis) during the body tilt maneuver (tilt angle shown by orange trace/right axis). The positive values indicate a rightward direction and the negative values indicate a leftward direction. vOCR, video ocular counter-roll.

We applied two different tilt maneuvers to account for the effect of the neck and head positions (Figure 1A). The head tilt maneuver was performed by applying a passive 30° lateral tilt of the head on the trunk while the subject was sitting upright. The body tilt maneuver was performed by applying a passive 30° lateral tilt of the head and trunk en bloc while the subject was sitting. In both the head and body tilt maneuvers, the tilt angle was measured by an accelerometer mounted on the VOG goggles and the examiner received numerical feedback in real-time to maintain a head pitch of 0° and a head roll of 30° during the maneuver. Per previous work, we chose a 30° lateral tilt as it is large enough to produce a significant OCR response ~5°, and yet within the comfortable range of positions to maintain during the head and body maneuvers for vOCR recording.^18,19^

During the test, subjects sat upright on a chair, fixing on a visual target (2.5 cm in diameter), 135 cm away at eye level, while their head was maintained in the upright position. The torsional eye position (ocular torsion) from both eyes as well as the head position was recorded simultaneously using the VOG goggles. First, a calibration was performed by setting the baseline references for the iris pattern, pupil position, and head position while the subject was sitting upright and looking straight ahead. These references were used to measure relative changes in the positions of the eye and head during the tilt maneuvers (details explained below under the VOG measurement). In each maneuver, the recording began with the head in the upright position for 30 seconds, followed by 3 right and 3 left lateral tilts in random order, each lasting 30 seconds, separated by 30-second periods with the head back to the upright position (Figure 1B). For the head tilt maneuver, subjects fixed on a visual target straight ahead throughout the task. For the body tilt maneuver, we used a soft collar to restrict the movement of the neck on the trunk. The soft collar was equipped with a protractor to ensure that the head and trunk were aligned and both tilted at 30° during the maneuver. In addition, instead of 1 target, 3 targets were used so that the straight-ahead fixation could be maintained during lateral tilts by instructing the subjects to fix on the lateral target along the side of the body tilt. All three targets were in line with the lateral targets placed at equal distances from the central target. The distance between the lateral and central targets was calculated as half of the distance between the sitting surface (subject’s hip level) to the canthus of the eye (ie, multiplied by the sine of 30°), hence taking into account the height of the trunk for maintaining straight ahead fixation during 30° body tilts.

#### vHIT Test

The standard vHIT maneuver was used to assess semicircular canal function, during which high-acceleration, small-amplitude passive lateral head rotations (“head impulses”) were applied in the yaw plane, that is, the plane of the horizontal canals. We used EyeSeeCam goggles and software (Interacoustics), which record horizontal and vertical eye and head movements simultaneously. Subjects sat upright on a chair, 135 cm away from a visual target. The movement of the head was recorded by inertial sensors on the goggles, and the movement of the eye was recorded by detecting the position of the pupil using an infrared camera, inserted above the right eye. First, the position of the eye was calibrated using laser lights on the goggles that provided fixation targets projected in front of the subject. These laser targets were used to measure the range of gaze positions (top, bottom, center, right, and left). To calibrate the head position, subjects were asked to keep their eyes on a stationary target straight ahead, while their head was passively rotated right and left (within the yaw plane) and then up and down (within the pitch plane). Following these calibration steps, subjects fixed on the target straight ahead, while lateral head impulses were delivered by the examiner from behind. The vHIT results were analyzed by the EyeSeeCam software in real-time, providing plots of accepted velocity waveforms for the head and eye movements within the range of 150 to 300°/s. Ten head impulses with successful eye and head recordings on each side were used by the software for vHIT measurements. All subjects underwent vOCR testing and vHIT, except 7 patients in the acute group in whom vHIT could not be performed due to the inconvenience of performing head impulses in the early stage of recovery following the surgery in these patients.

#### VOG Measurement

We used the RealEyes xDVR goggles (Micromedical Technologies Inc) for vOCR measurement. An accelerometer was mounted onto the goggles to measure the position of the head simultaneously (MPU-92/65 sensor with 3-axis gyroscope and accelerometer), which was connected through an Arduino board (Teensy 3.2) to the port of one of the cameras. We used custom software previously developed by our group to measure ocular torsion.^15^ This software uses template matching of the entire iris and also tracks the position of the eyelids to account for a partial occlusion of the iris and pupil.^15^ A polar transformation is applied to the iris pattern and the image is optimized to enhance the iris features, and mask the parts covered by the eyelids. A template matching method is then implemented to compare the current iris pattern at any given point in time with a reference image obtained at the beginning of the recording when the head is upright. Thus, all measurements of torsional eye position are relative to this reference image. Further technical details of this method have been previously published.^15^

### Data Analysis

#### vOCR

The vOCR responses were measured with the gain value calculated as the ratio of ocular torsion to head tilt position. The ocular torsion and head position data were analyzed in Matlab (Mathworks Inc). An average of 3 measurements in each tilt direction determined the final vOCR gain. The measurement of vOCR gain was done separately with the head and body tilt maneuvers and in each maneuver with lateral tilts to each side (ie, right and left). The static vOCR gain was measured as the median of vOCR gain values during the period after the head and eye reached the static position. To further examine the pattern of static and dynamic vOCR responses, we fitted an exponential curve to the gain responses over time in each subject:

$$y=\alpha (1-e^{\frac{-x}{\tau }}).$$

Here, x is the time and y is the vOCR gain, while α is the amplitude of the curve and τ is the time constant of the curve. Only gain responses that could be fitted with an exponential curve were included (90.1% of all tilts among subjects). The fitting was done from the time point when the head reached 20° from the upright position in all subjects. With this approach, the amplitude of the curve can provide a measure for the combined effects of dynamic and static vOCR gains, and the time constant can provide a measure for the transition between the dynamic and static phases of the vOCR gain.

#### vHIT

The average vHIT gains were calculated for 10 head impulses to the right side and 10 head impulses to the left side. This vHIT gain was calculated by the EyeSeeCam software as the eye velocity divided by the head velocity at 60 ms after the start of the head movement.

In order to compare vOCR and vHIT data, the corresponding asymmetry ratios were calculated in each patient, which accounted for the difference between the test values on the lesion side (ie, the side of vestibular loss or $T_{LS}$) and the nonlesion side $\left(T_{NLS}\right)$:

$$\text{Asymmetryratio}\;=\frac{abs\left(T_{NLS}\;-\;T_{LS}\right)}{T_{NLS}\;+\;T_{LS}}.$$

Here, the asymmetry ratio of zero means no difference between both sides, and larger asymmetry ratios indicate a larger difference between the lesion and nonlesion sides.

To compare vOCR gain values among subject groups, we conducted a 3-way repeated measures analysis of variance (3-way analysis of variance [ANOVA]) on the influence of 3 independent variables: Subject group (control, acute, subacute, and chronic groups; Table 1), vOCR maneuver (head tilt and body tilt), and the side tested (lesion/nonlesion sides in patients; 2 randomly assigned sides in controls).

To compare vHIT gain values, we conducted a 2-way repeated measures ANOVA between the 2 sides tested across the 4 subject groups. To compare vHIT and vOCR results, a 2-way repeated measures ANOVA of asymmetry ratios was used (vHIT, head-tilt vOCR, and body-tilt vOCR) across the patient groups (acute, subacute, chronic).

## Results

**Figure 2.**
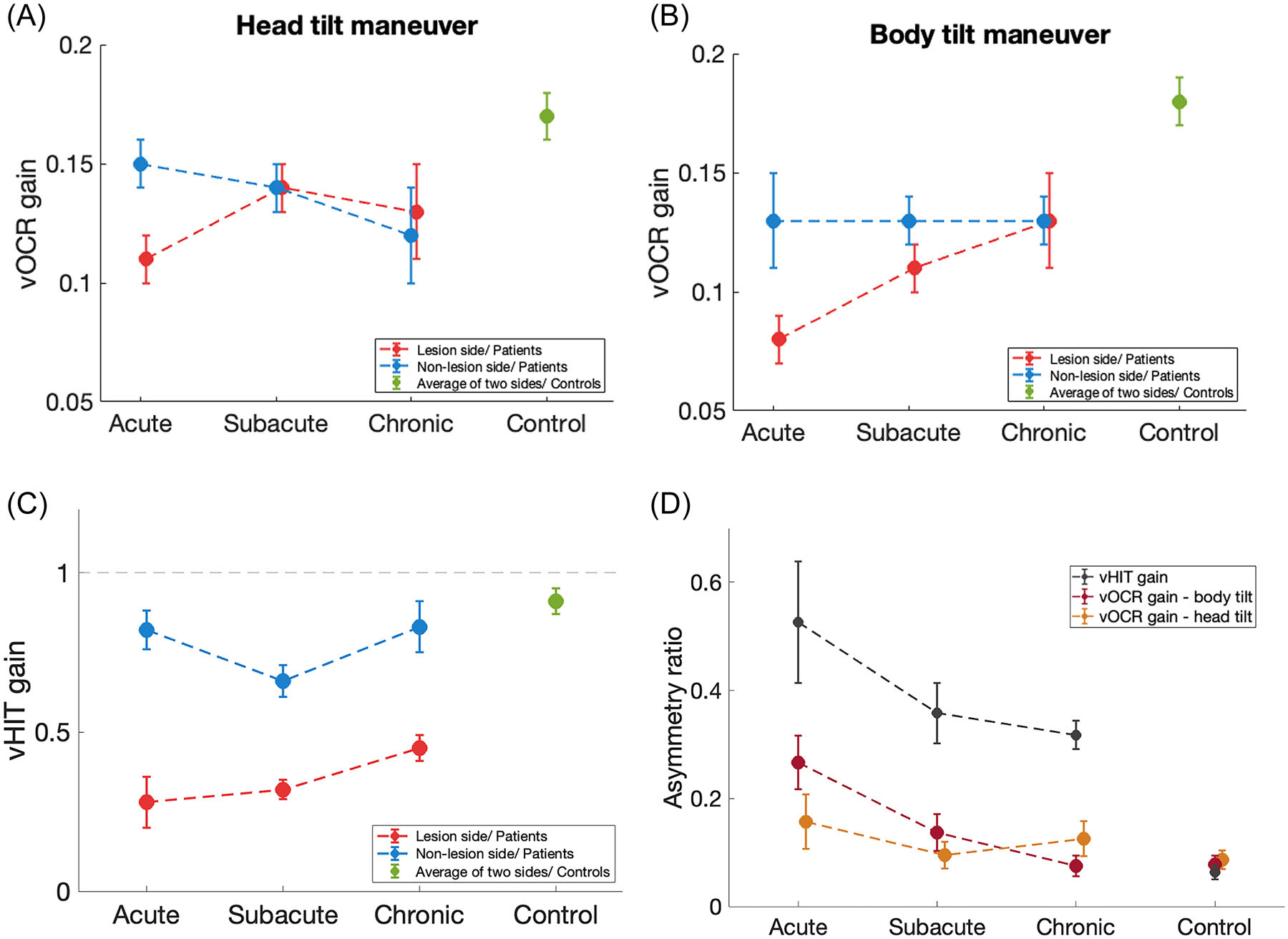
The mean vOCR gains for the head tilt (A) and body tilt (B) are shown along with the mean vHIT gains (C) and asymmetry ratios (D). Error bars represent SEM. Significant comparisons: (i) vOCR head-tilt; acute lesion side versus nonlesion side (*p* = .01) and lesion-side acute versus control (*p* = .02), (ii) vOCR body-tilt; acute lesion side vs nonlesion side (*p* = .001); lesion-side acute versus chronic (*p* = .048), acute versus control (*p* < .0001); subacute versus control (*p* = .004); nonlesion-side acute versus control (*p* = .016), subacute versus control (*p* = .03), chronic versus control (*p* = .03), (iii) vOCR head-tilt versus body-tilt; lesion-side acute (*p* = .01) and lesion-side subacute (*p* = 0.02), (iv) vHIT; lesion-side acute versus control, subacute versus control, chronic versus control (all *p* < .0001); acute lesion side versus nonlesion side (*p* < .00001), subacute (*p* < .00001), chronic (*p* < .00001). SEM, standard error of mean vHIT, video head impulse testing; vOCR, video ocular counter-roll.

The static vOCR gains for the head tilt and body tilt maneuvers are presented along with the vHIT gains for all subject groups in Table 2 and Figure 2. In the control group, neither the vHIT gains nor the vOCR gains were significantly different between the right and left sides (paired *t* test, *p* > 0.3). Thus, we used the average vOCR and vHIT values from both sides in the control group to compare with the lesion and nonlesion sides from the patient groups.

### Static vOCR

#### Static vOCR and Stage of Vestibular Loss

There was a significant difference in the vOCR gain between all subject groups (3-way repeated measures ANOVA; *p* = .003). Post hoc multiple comparisons showed that with the head tilt maneuver, the vOCR gain on the lesion side was significantly lower in the acute patient group compared with the control group, but there was no significant difference between the acute, subacute, and chronic patient groups (Figure 2A). No significant difference was found in the vOCR gain on the nonlesion side between all subject groups with the head tilt maneuver (Figure 2A).

With the body tilt maneuver, the vOCR gain on the lesion side in the acute patient group was significantly lower than the chronic patient and control groups, but it was not significantly different from the subacute patient group (Figure 2B). The vOCR gain was also lower on the lesion side in the subacute group compared with the control group, but it was not different from the chronic group (Figure 2B). No significant difference was found in the vOCR gain between the chronic and control groups on the lesion side (Figure 2B). On the nonlesion side, the vOCR gain in the acute, subacute, and chronic patient groups was lower than the control group, but there was no significant difference between the 3 patient groups (Figure 2B).

#### Static vOCR and Side of Vestibular Loss

There was a significant difference in the vOCR gain between the lesion and nonlesion sides (3-way repeated measures ANOVA; *p* = .005). There was also a significant interaction between the side of vOCR measurement and subject groups (*p* = .005). Post hoc multiple comparisons showed that with the head tilt maneuver, the vOCR gain on the lesion side was significantly lower than the nonlesion side in the acute patient group (Figure 2A). With the body tilt maneuver, the vOCR gain on the lesion side was also lower than the vOCR gain on the nonlesion side in the acute patient group (Figure 2B). No significant difference was found in the vOCR gain between the lesion-side and nonlesion-side in the subacute, chronic, and control groups with either the head-tilt or body-tilt maneuvers (Figure 2A and B).

#### Static vOCR and Tilt Maneuvers

There was a significant interaction between the maneuver type and subject group (3-way repeated measure ANOVA; *p* = .0017). Post hoc multiple comparisons showed that the vOCR gain on the lesion side was significantly higher with head tilt than with body tilt in the acute and subacute groups but not in the chronic group (Figure 2A and B). No significant difference was found in the vOCR gain on the nonlesion side between the head tilt and body tilt maneuvers in the acute, subacute, and chronic groups (Figure 2A and B).

#### vHIT

There was a significant difference in the vHIT gain between all subject groups (2-way repeated measures ANOVA; *p* < .00001), and also a significant interaction between the side of vHIT measurement and subject groups (*p* < .00001). Post hoc multiple comparisons showed that the vHIT gain on the lesion side in each patient group was significantly lower than the vHIT gain in the control group (Figure 2C). No significant difference was found in the vHIT gain on either the lesion or nonlesion sides between the 3 patient groups (Figure 2C). In addition, the vHIT gain on the lesion side was significantly lower than the vHIT gain on the nonlesion side in all patient groups (Figure 2C).

#### vOCR and vHIT Comparison

The vOCR and vHIT asymmetry ratios (range 0–1) from (Figure 2D and Table 3) were significantly different in all patient groups (2-way repeated measures ANOVA; *p* < .02). Post hoc multiple comparisons showed that the vOCR asymmetry with head tilt was significantly lower than the vHIT asymmetry in each patient group (Figure 2D). The vOCR asymmetry with body tilt was also significantly lower than the vHIT asymmetry in each patient group (Figure 2D). There was no significant difference between the vOCR asymmetries with head tilt and body tilt in any of the patient groups (Figure 2D).

#### vOCR Time Course

**Figure 3.**
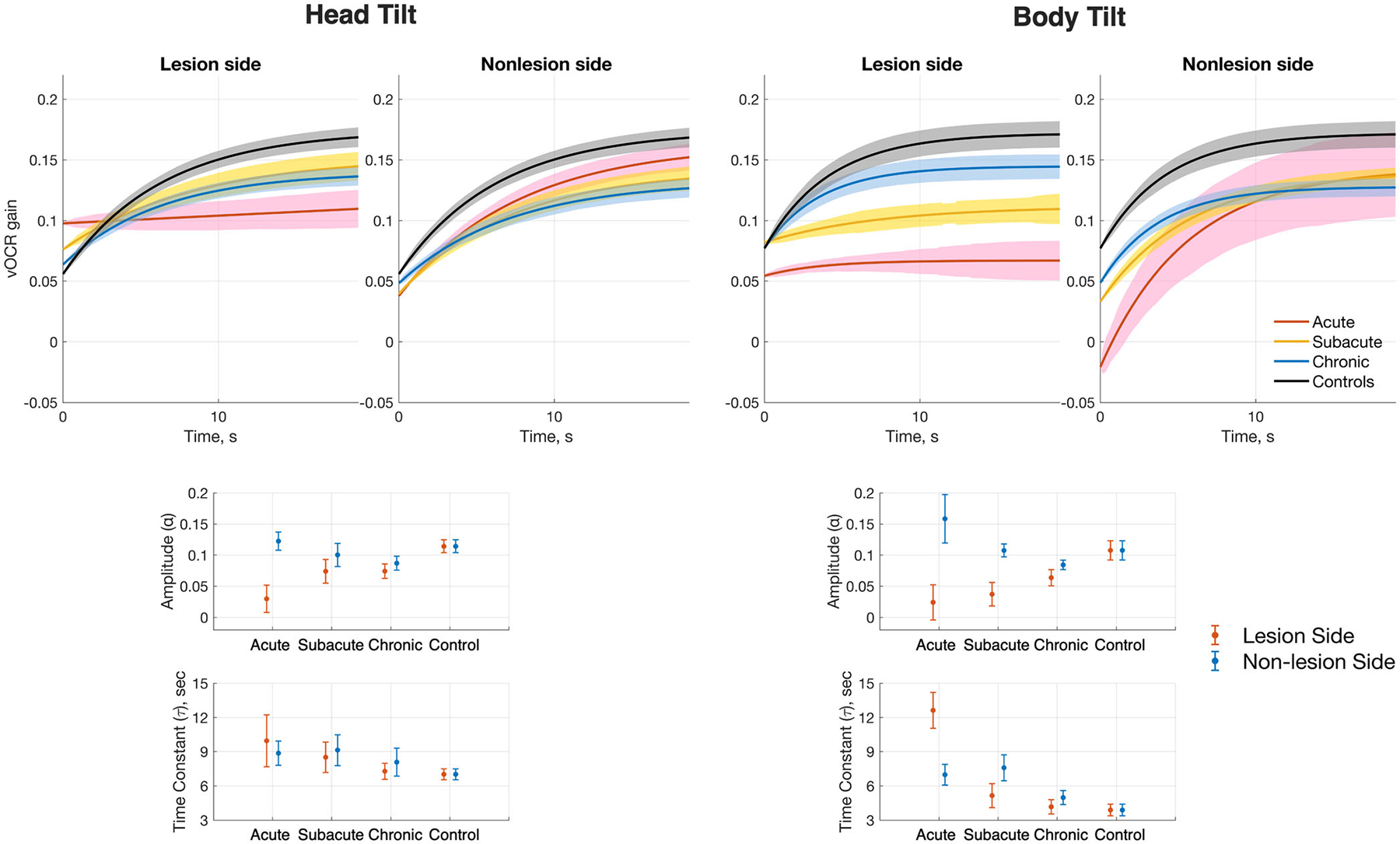
Exponential curve fits to the average time course of vOCR gains are shown (top panel) along with the fitting parameters (amplitude and time constant of, bottom panel). Both the head-tilt and body-tilt maneuvers are included starting with the vOCR gain at 20°. The shaded colors indicate SEM. Significant comparisons: (i) head-tilt amplitude; lesion-side acute versus control (*p* = .002), acute lesion side versus nonlesion side (*p* < .0001), (ii) body-tilt amplitude; lesion-side acute versus control (*p* = 0.03), acute lesion side versus nonlesion side (*p* < .0001), (iii) body-tilt time constant; lesion-side acute versus subacute, acute versus chronic, and acute versus control (all *p* < .0001), acute lesion side versus nonlesion side (*p* = .002). SEM, standard error of mean; vOCR, video ocular counter-roll.

The exponential fits to the average vOCR gain with their corresponding fitting parameters (amplitude and time constant) are shown for each subject group in Figure 3 and Table 4. There was a significant difference in the vOCR curve amplitude between the lesion and nonlesion sides (3-way repeated measures ANOVA; *p* < .00001). There was also a significant interaction between the side and subject group (*p* = .0001). Post hoc multiple comparisons showed that with the head tilt maneuver, the lowest curve amplitude on the lesion side was in the acute patient group, while the largest amplitude was in the control group (Figure 3). There was a significant difference in curve amplitude between the lesion and nonlesion side with the head tilt maneuver in the acute patient group but not in the subacute or chronic patient groups (Figure 3). There was a similar trend on the lesion side with the body tilt maneuver, which also showed the lowest curve amplitude in the acute patient group and the largest amplitude in the control group (Figure 3).

No significant difference was found in the curve amplitude on the nonlesion side with either the head-tilt or body-tilt maneuver in the acute, subacute, chronic, and control groups (Figure 3). With the body tilt maneuver, there was a significant difference between the curve amplitudes on the lesion and nonlesion side in the acute patient group, but not in the subacute or chronic patient groups (Figure 3).

There was also a significant difference in the time constant between the maneuver types (3-way repeated measures ANOVA; *p* < .0001) with significant interactions between the maneuver type and subject group (*p* = .036) as well as the side and subject group (*p* = .045). Post hoc multiple comparisons showed no significant difference in the time constant on either the lesion or nonlesion side with the head tilt maneuver between all subject groups (Figure 3). There was also no significant difference found in the time constant between the lesion and the nonlesion side with the head tilt manuever in any subject group (Figure 3).

With the body tilt maneuver, the slowest response on the lesion side (ie, largest time constant) was in the acute patient group (Figure 3). The time constant was higher on the lesion side than the nonlesion side in the acute patient group but not in the subacute or chronic patient groups (Figure 3).

## Discussion

In this study, we measured vOCR using simple head and body tilt maneuvers. Our results show vOCR responses evolve at different stages of recovery following vestibular loss. The gain of vOCR during static tilt was reduced in the acute stage of vestibular loss compared with the chronic stage. The curve amplitude of vOCR gain was also reduced in the acute stage suggesting that the dynamic phase during head movement was also affected by the loss of both otolith and canal functions in these patients. The time course of vOCR was affected as well, and it was longer in patients with acute vestibular loss, showing a slower response related to dynamic and static phases of vOCR.

Although the static vOCR response was reduced on the side of vestibular loss with both head and body tilts, the deficit was more pronounced with the whole body tilt. The contribution of neck inputs improved the gain of vOCR when the head was tilted on the body, and there was no significant difference between the early or late stages of vestibular loss. These findings suggest that the effect of neck improved otolith-ocular function following vestibular loss. The neck inputs also enhanced the time course of vOCR in patients with acute vestibular loss. These findings suggest a compensatory effect of neck proprioception on both the static and dynamic phases of vOCR response.

Following loss of vestibular function, patients may adopt different strategies to achieve compensation with other sensory information.^3^ Our results show the proprioceptive neck inputs can be part of this recovery process and compensate for loss of VOR in the roll plane. In line with this finding, de Graaf et al, in a small study found a similar enhancing effect of neck inputs in patients with vestibular hypofunction.^14^ When the trunk was tilted alone, the cervico-ocular response was reduced suggesting that any contribution from neck proprioception was mainly synergic to vestibular inputs, and it could not by itself generate a significant ocular response. There was no significant effect of neck inputs in healthy controls, and the roll VOR did not change neither when the whole-body was tilted, nor when the head was tilted on the body or when the trunk was tilted under the head.^13,14^ These findings are in line with our present results and overall show that the neck inputs can have an enhancing effect on the roll VOR in patients with loss of vestibular function. The compensatory effect of neck proprioception following vestibular loss is also shown for semicircular canals.^5^ This effect, however, is not enough to generate a compensatory VOR during high-frequency head movements, but it may have a role in triggering saccades at the onset of head motion to improve upon the inadequate VOR function.

Under normal circumstances, otolith inputs are in a state of equilibrium similar to the balance between the semicircular canals. With vestibular loss on one side, however, this natural set point can be disrupted and pathologically affect vestibular responses on both sides. Our results with reduced static vOCR gains on the nonlesion side are in line with this effect and show how compensatory mechanisms may also affect the gain on the healthy side. The time course of response was affected as well, with an overall increased amplitude of vOCR curve on the nonlesion side in patients with acute vestibular loss. This finding suggests that early compensatory mechanisms can enhance vOCR response on the healthy side. Interestingly, neck inputs could also modulate this effect, as the head-on-body tilt reduced the amplitude of vOCR curve on the healthy side in patients with acute vestibular loss.

Following vestibular loss, each patient may have a distinct strategy to compensate for aberrant vestibular information resulting in different functional outcomes. It is therefore key to detect such compensatory strategies and be able to enhance their outcomes through effective rehabilitation protocols.^3,26^ In this context, vOCR deficits correspond with the stage of vestibular loss and might be useful as a novel clinical marker to measure vestibular recovery or evaluate the compensatory effect of neck inputs in individual patients. To further examine these effects, future studies are needed to address whether vOCR measurements at different stages of vestibular loss correlate with the improvement of vestibular symptoms or overall functional recovery in these patients.

Our findings suggest that combining vHIT and vOCR measurements can be clinically valuable in patients at different stages of vestibular loss. Similar to vHIT, vOCR measurement can be performed as a bedside test using high-speed VOG goggles with automated analysis software.^18^ Based on our measurements, both vOCR and vHIT showed asymmetry with decreased values on the side of vestibular loss in acute patients. The vOCR asymmetry, however, was reduced in patients with subacute and chronic vestibular loss, while the vHIT asymmetry persisted in these patients. This finding is in line with previous studies that found minimal recovery in VOR measurements of canal function after vestibular loss, especially in those patients who had vestibular nerve resections.^27-29^ Despite this inadequate recovery in VOR gain, there is often functional improvement in patients over time, which suggests that a modest recovery within the low-frequency range of vestibular function may be significant for recovery. Other nonvestibular mechanisms such as how the brain can effectively generate compensatory saccades (eg, decreased latency or amplitude) or use neck proprioception to substitute for vestibular deficit may also largely contribute to functional improvements in patients.^5,29^

In this study, we used a separate group of patients at different stages of vestibular loss to compare our vestibular measurements. Future studies will have to examine vOCR measurements in the same group of patients over time in order to address whether vOCR recovery can be associated with improvement of daily function and vestibular symptoms in these patients. With such correlations, vOCR can be clinically valuable to track recovery in addition to detecting loss of otolith-ocular function.

In conclusion, our results suggest that vOCR deficits can recover following loss of vestibular function. In this process, neck inputs can enhance the vOCR response at different stages of vestibular loss. These findings have valuable implications for using vOCR as a clinical marker to detect the time course of vestibular deficit, measure vestibular recovery, or assess the compensatory effect of neck proprioception in patients with loss of vestibular function.

## Figures and Tables

**Table 1.** Demographics of Patient Subgroups and Healthy Control.

	Time from the lesion (d, mean ± SEM)	Number (female)	Age mean (SD)

Acute	≤4 wk (9 ± 2)	13 (6)	54.2 (10.8)
Subacute	4 wk to 6 mo (61 ± 11)	13 (7)	52.8 (13.2)
Chronic	> 6mo (1009 ± 266)	15 (8)	54.5 (11.3)
Control	N/A	15 (7)	52.5 (11.4)

Abbreviations: N/A, not applicable; SD, standard deviation; SEM, standard error of mean.

**Table 2.** Static vOCR Gains and vHIT Gains (Mean ± SEM) in All Patient Groups and Healthy Controls.

Test variable	Side	Acute	Subacute	Chronic	Control^a^

vOCR gain, head tilt	Lesion Nonlesion	0.11 ± 0.01 0.15 ± 0.01	0.14 ± 0.01 0.14 ± 0.01	0.13 ± 0.02 0.12 ± 0.02	0.17 ± 0.01
vOCR gain, body tilt	Lesion Nonlesion	0.08 ± 0.01 0.13 ± 0.02	0.11 ± 0.01 0.13 ± 0.01	0.13 ± 0.02 0.13 ± 0.01	0.18 ± 0.01
vHIT gain	Lesion Nonlesion	0.28 ± 0.08 0.82 ± 0.06	0.32 ± 0.03 0.66 ± 0.05	0.45 ± 0.04 0.83 ± 0.08	0.91 ± 0.04

Abbreviations: SEM, standard error of mean; vHIT, video head impulse testing; vOCR, video ocular counter-roll.

a The mean values from the 2 sides are provided for the control group.

**Table 3.** vOCR Gain and vHIT Gain Asymmetry Ratios (Mean ± SEM) in All Patient Groups.

Asymmetry ratio	Acute	Subacute	Chronic

vOCR gain, head tilt	0.16 ± 0.05	0.09 ± 0.03	0.13 ± 0.03
vOCR gain, body tilt	0.27 ± 0.05	0.14 ± 0.03	0.07 ± 0.02
vHIT gain	0.53 ± 0.11	0.36 ± 0.06	0.32 ± 0.03

Abbreviations: SEM, standard error of mean; vHIT, video head impulse testing; vOCR, video ocular counter-roll.

**Table 4.** Fitting Parameters (Mean ± SEM) of vOCR Curve Fitting Including Amplitude (*α*) and Time Constant (*τ*) for All Patient Groups and Healthy Controls.

Patient	Side	Head tilt		Body tilt	
		*α*	*τ*, s	*α*	*τ*, s

Acute	Lesion	0.03 ± 0.02	9.95 ± 2.28	0.02 ± 0.03	12.62 ± 1.57
	Nonlesion	0.12 ± 0.01	8.86 ± 1.07	0.16 ± 0.04	6.98 ± 0.90
Subacute	Lesion	0.07 ± 0.02	8.51 ± 1.33	0.04 ± 0.02	5.15 ± 1.04
	Nonlesion	0.10 ± 0.02	9.13 ± 1.36	0.11 ± 0.01	7.58 ± 1.12
Chronic	Lesion	0.07 ± 0.01	7.28 ± 0.71	0.06 ± 0.01	4.17 ± 0.63
	Nonlesion	0.09 ± 0.01	8.08 ± 1.23	0.08 ± 0.01	4.98 ± 0.62
Control		0.11 ± 0.01	7.01 ± 0.48	0.11 ± 0.02	3.89 ± 0.50

Abbreviations: SEM, standard error of mean; vOCR, video ocular counter-roll.
